# Atypical maternal interaction is associated with elevated levels of hair cortisol in children

**DOI:** 10.3389/fped.2022.994882

**Published:** 2023-01-25

**Authors:** Franziska Köhler-Dauner, Inka Mayer, Melissa Hitzler, Alexander Karabatsiakis, Lynn Matits, Alexandra M. Bach, Katharina Rost, Jörg M. Fegert, Iris-Tatjana Kolassa, Ute Ziegenhain

**Affiliations:** ^1^ Department of Child and Adolescent Psychiatry/Psychotherapy, Ulm University, Ulm, Germany; ^2^ Clinical & Biological Psychology, Institute of Psychology and Education, Ulm University, Ulm, Germany; ^3^ Department of Psychology, Clinical Psychology II, University of Innsbruck, Innsbruck, Austria; ^4^ Division of Sports and Rehabilitation Medicine, Department of Medicine, Ulm University Hospital, Ulm, Germany

**Keywords:** mother-child interaction, chronic stress, endocrine stress response, hair cortisol concentrations (HCC), intergenerational transmission

## Abstract

The quality of maternal caregiving is an important factor in the healthy development of a child. One consequence of prolonged insensitive and atypical maternal interaction behavior (e.g., withdrawing from interactions with the child and role-reversal, i.e., the takeover of the parental role or parts of it by the child) in mother-child-dyads can cause alteration of the child's stress response system. Higher salivary cortisol concentrations were reported in infants and toddlers directly after negative interactions with their parents. However, no study to date has examined the association between atypical maternal interaction behavior and hair cortisol concentrations (HCC) in infants. Here, we studied the association of maternal interaction behavior with HCC of the child. Mother-child dyads (*N *= 112) participated in the longitudinal study *My Childhood—Your Childhood*. The *AMBIANCE* scale and its subscales were used to assess atypical maternal interaction behavior during the Strange Situation Procedure. Chronic stress levels in the child were assessed by HCC of 3 cm hair strands at the age of 12 months. Maternal educational level (operationalized in highest education level) served as a control variable. Robust multiple linear regression analyses revealed that role/boundary confusion was associated with HCC, i.e., the higher atypical interaction behavior of the mother the higher the HCC in the children. By measuring hair cortisol in this study, it is possible to determine the average long-term activity of the child's stress response system.Thus, atypical maternal interaction behavior could be a risk factor for persistent stress in children, contributing to a higher risk for negative health outcomes in later life. The results of this study highlight the importance of early intervention programs that focus on the relationship between mother and child.

## Introduction

The family environment is a crucial influencing factor for the healthy development of a child (1, 2). Particularly, the interaction of parents and other caregivers with the infant has a major impact on child development such as attachment representation (3–6), receptive language development (7), emotion regulation (8, 9) or the risk for mental disorders later in life (10, 11). Indeed, atypical maternal behavior such as withdrawing from interactions with the child or role-reversal, which means the takeover of the parental role or parts of it by the child was linked to disorganized attachment patterns among infants and toddlers (5, 6, 12, 13). The kindergarten-aged children showed poorer receptive and expressive language skills when their mothers behavior was more intrusive (7). A longitudinal study indicated that especially young adults with less responsive parents in childhood reported dissociative symptoms compared to children with more responsive parents (10). Moreover, low quality of parental interaction behavior in childhood was associated with physical diseases such as inflammatory bowel disease (14), asthma, respiratory infections (15–17), migraine (17, 18), and allergies (15).

A paternal lack of sensitive and appropriate responses to the child's emotions and behaviors is a chronic psychosocial stressor and might chronically stimulate the hypothalamic-pituitary-adrenal (HPA) axis and lead to a release of cortisol from the adrenal cortex into the bloodstream (19–22). Indeed, chronic psychosocial stress has been linked to allostatic overload of the body's stress response systems and an increased risk of physical and mental disorders (23). Smeekens and colleagues (24) found a stronger increase in 5-year-olds' salivary cortisol concentrations following a negatively attributed interaction with parents. Particularly children with low ego resilience, i.e., a decreased ability to cope appropriately with stress, displayed a stronger stress response in negative interactions with their parents (24). Furthermore, in a sample of mothers with a post-partum anxiety disorder a correlation between the mother's stress level during pregnancy and the baby's cortisol response has been reported (25).

An important factor contributing to elevated mental stress levels and cortisol levels in adulthood is experiencing abuse and neglect during childhood. It has been assumed that intergenerational transmission of childhood maltreatment (CM) experiences from the mother to her child is found because appropriate regulatory and coping strategies were not developed (26). Another impact of CM on the next generation, considering the findings of Zietlow and colleagues (25), could also be an atypically increased salivary cortisol concentration and reactivity of the child as a consequence of the Face-to-Face-Still-Face paradigm (FFSF). Furthermore, maternal HCC has been associated with infant salivary cortisol levels when the mothers were more intrusive and had lower positive engagement synchrony in the interaction with their infants (27). Thus, there appears to be an interrelation between the stress response systems of mother and child.

Negative interactions between mother and child occur as a chronic stressor. Therefore, the child's brain might be exposed to chronically elevated levels of cortisol, which may result in increased HCC in the child (19, 21). A study looking at chronic biological stress levels of parents and preschoolers found a moderation of the association between parental and child HCC by emotion regulation skills of the child (28). Meaning, children with good emotion regulation abilities were not as affected by intergenerational transmission effects of chronic stress. Furthermore, such children showed less strong associations between parental financial status and HCC. In contrast, children with less developed emotion regulation were found to have an increased HCC, which was negatively associated with the socioeconomic status (SES) of the family. Vaghri and colleagues (29) also found a stable negative correlation between HCC in children and parental education levels. This might be due to the exposure to various social stressors because of lower financial resources. Thus, consideration of those variables in the context of cortisol research is important.

To conclude, atypical maternal interaction patterns with the child can act as psychosocial stressors negatively affecting its development as well as mental and physical health in later life. However, to our knowledge, no study so far investigated the relationship between atypical maternal behavior and the child's neuroendocrine stress response measured *via* HCC. The aim of this study was to fill this gap. We hypothesized that higher atypical maternal interaction behavior (operationalized by the *Atypical Maternal Behavior Instrument for Assessment and Classification* [AMBIANCE] (30); would be positively associated with higher HCC of infants.

## Methods

### Study design and recruitment of participants

Within the project TransGen, mother and infant dyads were investigated longitudinaly starting from birth. The recruitment took place in the women's hospital of the University hospital of Ulm shortly after parturition. The study was financially supported by the German Federal Ministry of Education and Research BMBF and approved by the local Ethics Committee of Ulm.

The recruitment of mother-child-dyads (*N* = 533) started in October 2013 within 1 to 6 days after parturition (timepoint t_0_). Mothers with twins were not recruited for the study. At t_0_, mothers provided written informed consent before participating in the study. They then took part in an initial screening interview. CM load was assessed using the *Childhood Trauma Questionnaire* [CTQ, (31)] including emotional, physical and sexual abuse as well as physical and emotional neglect. All mother–child-dyads were invited at three sequential time points: 3 months (*t*_1_), 12 months (*t*_2_), and 24 to 36 months after birth (*t*_3_). The following data regarding the *Atypical Maternal Behavior Instrument for Assessment and Classification (AMBIANCE)* scale (30) and HCC of infants were assessed at *t*_2_. Likewise, depressive symptomatology was assessed with the Brief Symptom Inventory [BSI, (32)]. For data collection, all mother-child-dyads were invited from 10 a.m. to 1 *p*.m. to the Department of Child and Adolescent Psychiatry/Psychotherapy at the Ulm University Hospital. During their visit, mothers and children were first asked to listen to a calming digital lullaby (episode 1). Afterwards, all mother-infant-dyads performed the *Strange Situation Procedure*, for which the AMBIANCE was than later coded.

### Participants

In total, *N *= 112 mother-child-dyads participated at both time points *t*_0_ and *t*_2_. One person had to be excluded from the sample due to taking cortisone medication. The average age of participating mothers was *M *= 34 years (*SD *= 5), ranging from 21 to 45 years. Considering educational qualifications (measured as an ordinal variable with a range from 2 = “lowest school education level” to 5 = “*highest school education level”*), 71.9% of all mothers reported a high school diploma, whereas 21.6% had a secondary school diploma, and 6.6% had a lower secondary diploma as the highest education certificate. The mean *T*-value measured with the BSI at time point *t*_2_ was *M *= 46.71 (*SD *= 8.42). *n *= 6 of the mothers from this study reported depressive symptoms in the conspicuous range with *T* > 60. The average age of all infants was *M = *53.22 weeks (*SD *= 5.25) with their ages ranging from 44 to 78 weeks. Almost half of all infants (48.2%) were males.

## Measures

### AMBIANCE scales

All Strange Situation Procedure sessions were videotaped to analyze the quality of maternal interactive behavior between the mother and her infant using the *AMBIANCE* (30). Lyons-Ruth and colleagues (33) based the development of the *AMBIANCE* instrument on Main and Hesse's theory, which explains the frightened, frightening, and dissociated parental behavior (34, 35). Therefore, they considered profound disruptions in mother-child interactions and emotional as well as physical withdrawal behaviors as anomalous parental behavior of mothers during the interactions with their children (36). The *AMBIANCE* is a coding system that assesses disrupted maternal behaviours on five dimensions on a 7-point scale: (1) affective communication errors, (2) role/boundary confusion, (3) disorganized/ disoriented behaviors, (4) negative/intrusive behavior, and (5) withdrawal. For a final assessment, an overall score of the general level of disruption is determined. This score is based on the displayed level of intensity and frequency of disrupted behaviors during the recorded mother-child-interaction, whereby a level of disrupted communication of up to 4 is considered “not-disrupted” and a level of 5–7 is considered “disrupted”. A single coder, who was trained by and reliable according to the original developers of the *AMBIANCE*, scored all play sessions blinded to the data sets of the mother-infant-dyads (30).

### Hair cortisol concentration (HCC)

At *t*_2_, hair strands of mothers and their children were collected from the posterior vertex region of the scull, preferably cut as close as possible to the scalp. Following the recommendations of the Society of Hair Testing, two to three hair strands with a diameter of at least 3 mm were collected to have sufficient hair material for the subsequent biolaboratory analyses (37, 38). Under sterile conditions, the hair samples were wrapped with aluminium foil and stored at −20°C until preanalytical processing. Preprocessing of all hair samples was performed in the laboratory of Ulm University. To avoid contamination of the hair with skin moisture, hair samples were processed using laboratory gloves. Hair strands of the same subject were pooled. From the proximal end, hair strands were cut into 3 cm long segments to assess HCC of the last 3 months prior to sampling. According to a study by de Kruijff and colleagues (39) childrens hair grows approx. 1 cm/month already at this age. Hair segments were then placed into cryotubes with a standardized hair weight (range 4–6 mg) per sample. Samples were shipped to the laboratory of Prof. Dr. Kirschbaum at the Technical University of Dresden in Germany for analysis. With reference to the protocol of Gao and colleagues (40), every 3 cm hair segment was washed with isopropanol twice and dried for at least 12 h under a stream of nitrogen gas. Subsequently, cortisol was extracted by placing the same non-pulverized hair segment in a methanol solution at room temperature overnight. The supernatant which remained after centrifugation (10,000 rpm for 2 min) was used, while the methanol was evaporated at 65°C under a constant nitrogen stream. All dried samples were then resuspended in 150 μl double-distilled water. Mass spectrometry-analysis of HCC was conducted using a Shimadzu HPLC system (Shimadzu, Canby, OR) coupled to an ABSciex API 5,000 Turbo ion-spray triple quadruple platform (AB Sciex, Foster City, CA) as reported previously (38).

### Statistical analyses

Data were analyzed using R version 4.2.1 (41) and *p*-values ≤ 0.05 were considered as significant. Due to the skewed distributions of HCC data, the natural logarithm was applied and logarithmized data were used in further analyses. In advance, boxplots were used to identify potential outliers. Three potential outliers were identified (at least > 2 *SD*). The outliers showed no physiological or medical reason for exclusion. However, adding or removing these data points did not change the pattern of results and they were therefore retained in the analyses.

For multiple regression models, assumptions of linear regression were checked visually using scatter plots, standardized residuals, and leverages (to check for linearity as well as for outliers) and P-P plots (to check for normal distribution of the residuals). The tolerance and variance inflation factor values were used to check for multicollinearity.

Due to skewness and outliers in HCC data, the use of traditional parametric methods was considered inappropriate. Thus robust multiple linear regression models [R package: robust, (42)], with HC4 estimators for heteroscedasticity were calculated to test the second hypothesis. Due to theoretical overlap and intercorrelation of the *AMBIANCE* subscales with the *AMBIANCE* global score, two separate robust regression models were calculated. We used AIC and BIC for model selection to distinguish among a set of possible models describing the relationship between HCC and the *AMBIANCE* global score (model 1), and respectively the five *AMBIANCE* subscales (model 2). The two models with the best model-to-data fit were selected for analysis. For all analyses, maternal age and education level (categorical variable: high school diploma, secondary school diploma, lower secondary diploma) and child's sex were considered as additional covariates. The best fit for model 1 included the *AMBIANCE* global score and mother's education as covariate. The best fit for model 2, regarding the *AMBIANCE* subscales included: role/boundary confusion, negative/intrusive behavior and mother's education as covariates.

## Results

### Descriptive analyses

The descriptive statistics of the *AMBIANCE* global score, its subscales and HCC are provided in Table 1.

**Table 1:** Descriptive statistics of AMBIANCE subscales, AMBIANCE global score, and child's hair cortisol for *N *= 111 mother-child-dyads.

	*M*	*SD*	*Min*	*Max*
**Age of mother (in years)**	33.9	4.3	25.4	44.3
**Age of children (in weeks)**	53.22	5.25	44.00	78.00
**AMBIANCE scales**				
Affective communication errors	3.1	1.3	1	6
Role/boundary confusion	2.4	1.5	1	6
Fearful/disoriented behavior	2.6	1.4	1	6
Intrusive/negative behavior	2.3	1.2	1	5
Withdrawal	3.1	1.3	1	6
Global score	3.6	1.2	1	6
**Hair cortisol child (pg/mg)**	359.6	275.7	30.9	1600.7

### Multiple linear regression

**Figure 1:**
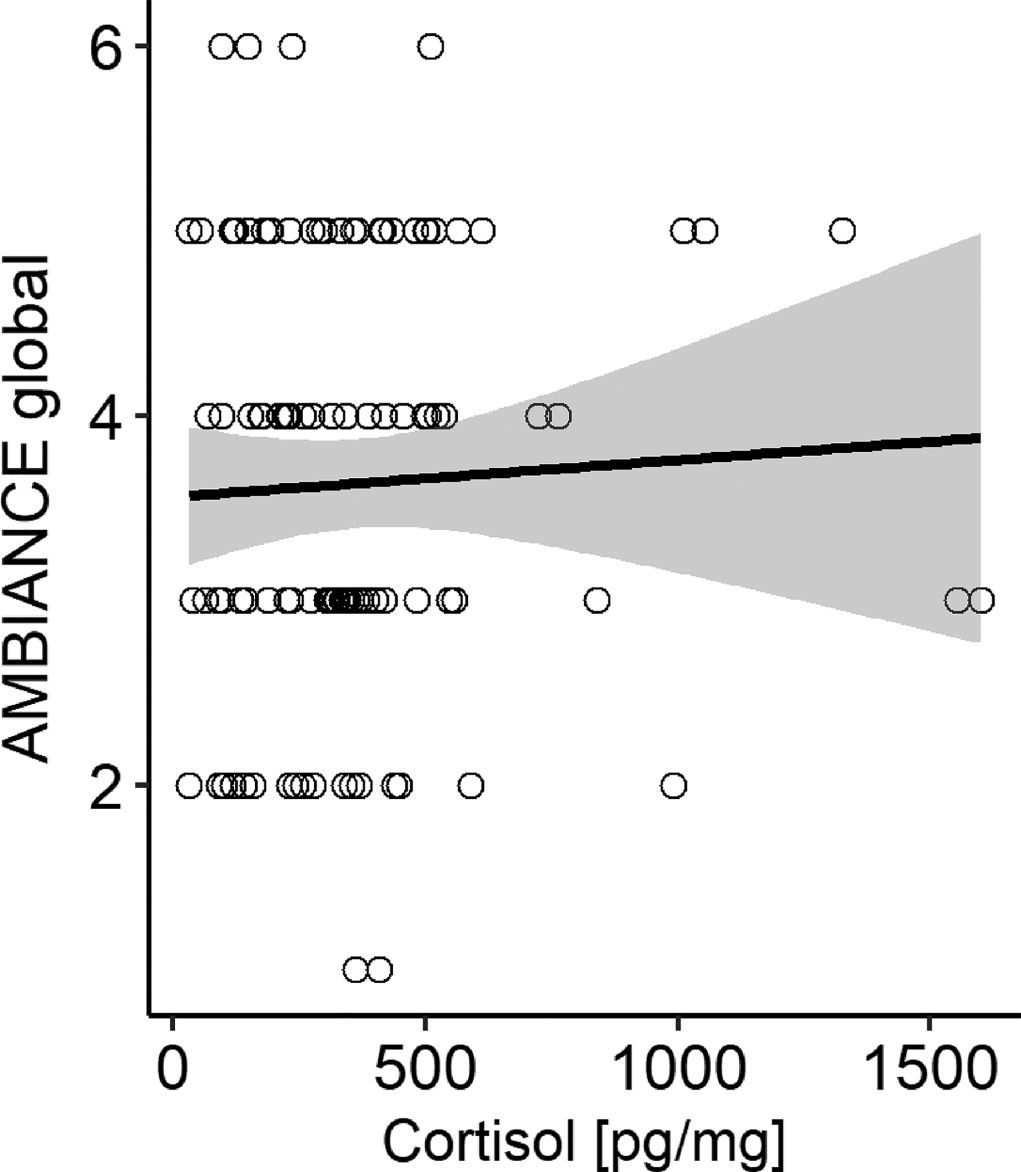
Scatterplot with HCC raw data for robust regression model including AMBIANCE global score as predictor. Grey areas depict the 95% confidence interval.

**Figure 2:**
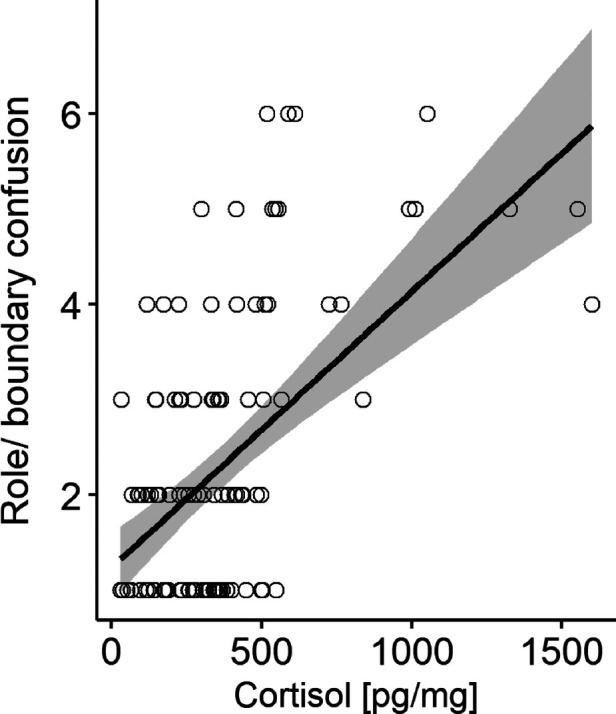
Scatterplot with HCC (pg/mg) raw data for robust regression model including AMBIANCE scale role/boundary confusion as predictor. Grey areas depict the 95% confidence interval.

The results of the two multiple linear regression models are shown in Tables 2, 3 and illustrated in Figures 1, 2.

**Table 2:** Results of robust multiple linear regression with AMBIANCE global score as predictor for child's hair cortisol level and maternal education as covariate.

Model	*b (SE)*	*ß*	*p*	*95%-CI*
***Children's hair cortisol***				
* Constant*	6.27 (0.40)	.05	<.001***	[5.34, 7.17]
* AMBIANCE global score*	−0.005 (0.06)	−.01	.952	[−0.14, 0.13]
* Education level of the mother*	−0.14 (0.07)	−.18	.103	[−0.29, 0.03]

***: *p *< .001; two-tailed significance testing; *p*-values adjusted with FDR (false discovery rate) for multiple comparisons; CI, confidence interval.

**Table 3:** Results of robust multiple linear regression with AMBIANCE subscales role/boundary confusion and intrusive/negative behavior as predictors for child's hair cortisol level and maternal education.

Model	*b (SE)*	*ß*	*p*	*95%-CI*
***Children's hair cortisol***				
* Constant*	5.45 (0.31)	.09	<.001***	[4.92, 5.94]
* Role/boundary confusion*	0.19 (0.05)	.37	<.001***	[0.14, 0.32]
* Intrusive/negative behavior*	0.08 (0.06)	.13	.199	[−0.08, 0.16]
* Education level of the mother*	−0.09 (0.07)	−.12	.177	[−0.20, 0.01]

***: *p *< .001; two-tailed significance testing; *p*-values adjusted with FDR (false discovery rate) for multiple comparisons; CI, confidence interval.

In the first model, the *AMBIANCE* global score was a predictor, while mother's education level was entered as a covariate: the overall model fit was not significant [*F*(2,108) = 1.92, *p *= .152], i.e., the model did not explain the data better than a simple overall mean model. This was also reflected in the explained variance of childrens' HCC levels which was only around 4%.

In Model 2, the subscales of the *AMBIANCE* “role reversal” and “intrusive/negative behavior” were entered as predictors for infants HCC, while mother's education was included as a covariate. The overall model was significant [*F*(3,107) = 12.21, *p *< .001] and explained 24% of the variance. Only the factor “role/boundary confusion” (*p *< .001) significantly predicted infants HCC.

## Discussion

This study investigated the association of various atypical maternal interaction patterns (as measured with the AMBIANCE global score and its subscales) with the children's HCC one year after birth. While the AMBIANCE global score was not associated with infant's HCC, the subscale “role/boundary confusion” in the AMBIANCE, was associated positively with higher HCC in children, i.e., the more inappropriate mother's behaviour in this subscale, the higher the HCC of the child. Therefore, this type of atypical maternal behavior might be a psychosocial stressor in early childhood linked to an altered cortisol secretion, endangering childrens mental and biological development and health. However, other explanations are equally plausible as the behaviour of mothers and the HCC of children could be influenced by a third unknown variable such as biological factors [e.g., higher levels of inflammation due to shared environment such as diet, living context (living close to busy streets with high levels of pollution, lack of green space), a pro-inflammatory diet, etc.] which might translate in atypical maternal interaction behaviour and higher HCC in children. Unfortunately, we did not directly assess the level of inflammation in our sample and cannot completely rule out inflammation as one factor underlying this association.

The association between more role confusing maternal behavior and higher HCC in children might indicate that this form of atypical interaction of the mother represents a psychosocial stressor that contributes (at least partially) to an increased biological stress response in the child. As HCC is a measure of chronic stress hormone accumulation over the last three months, this might suggest that these behaviors of the mother were not exclusively displayed in the artificial examination situation but are displayed regularly in everyday life. This interpretation could be in line with previous studies, which showed a correlation between inadequate interaction as well as cortisol levels of the mother in saliva and hair with the salivary cortisol of the child (24, 27).

Role reversal is described as the transfer of parental roles and functions to the child that should be addressed/fulfilled by the mother's partners or friends (43). More specifically, this means that children take over the care of the mother's needs (44). Macfie and colleagues (44) consider this a major risk for healthy child development. Indeed, if role reversal is present it can lead to the development of various psychopathologies later in life (45, 46). Parents with adverse childhood experiences are particularly prone to this kind of atypical interaction as they often show difficulities maintaining functioning relationships with peers (47). Thus, they try to meet their needs with the help of their child and seek the missing emotional support within this relationship. In the long run, if the mother's demands exceed the child's psychosocial and emotional resources, this can impede the child's psychosocial and emotional development (44). Hence our results emphasize atypical maternal interaction patterns, especially role reversal, as a critical psychosocial stressor presumably contributing to elevated stress levels in children. Consequently, this might partially contribute to permanently altered HCC and possibly also to higher inflammation levels in children. This influence seems to be stronger than the influence of maternal education as this factor was not significant in the regression models.

We observed no correlation of maternal age with any *AMBIANCE* score in the present study. In contrast, previous studies observed that the children of older mothers had on average lower HCC levels (48). Similarly, while previous studies found an influence of education on HCC of children (28), we observed no such association.

In contrast to our hypotheses, the subscales *withdrawal, fearful/disoriented behavior* and the global score of the *AMBIANCE* were not significantly correlated with HCC and were no significant predictors for infant's HCC. However, in a study by Khoury and colleagues (49), a correlation was found between maternal HCC and specifically increased withdrawal behavior but only when the mother also reported increased severity in depressive symptoms. It is possible that withdrawal behaviors represent a particular variation in the interaction, as Khoury et al. (49) found that only in individuals with severe depression there was a significant correlation between withdrawal behaviors and maternal HCC. In addition, the other subtypes of atypical interaction (e.g., role/boundary confusion) might induce stronger psychosocial and biological stress responses in the child and interfere more with its emotional regulation as compared to *withdrawal* or *fearful/disoriented behavior*, as there might be more intrusive/erratic behavior displayed by the mother than in passive withdrawal. In contrast, if mothers show inappropriate externalizing or unpredictable behavior towards their child, a withdrawal by the mother might be experienced as a short-time relief. As a consequence, the child could reorient without being confronted with repeated potential stressful interactions.

### Limitations and future direction

Our study cohort comprised predominantly healthy women in the postpartum period, living mostly in a committed relationship, with a high level of education, and relatively high socioeconomic status. Atypical maternal interaction behavior in our healthy community sample was comparably low, and—given the relatively high SES, this distribution might underestimate the effect sizes of atypical interactions on HCC. An intentional oversampling of at-risk mothers could be a solution in future studies. Therefore it should be kept in mind when applying the results to other cohorts and contexts that the generalizability might be limited. Nevertheless, social desirability effects were avoided in the classification of the interactions as this was based on external raters and not on self-assessment by the mothers themselves. In future studies additional relevant factors should be considered such as children's and parents’ level of inflammation and environmental factors contributing to a pro-inflammatory state in the body as these factors might have an influence on cortisol. In addition, breastfeeding should be considered in future studies, as findings from previous studies show that breastfeeding is associated with lower cortisol levels in children [e.g., (50)].

## Conclusion

We found inappropriate parenting behavior, more specifically role/boundary confusion, to be associated with higher HCC in the children. By measuring hair cortisol in this study, it was possible to determine the average long-term activity of the child's stress response system, therefore the increased HCC might indicate an increased state of allostatic load in the affected children, which might create a higher risk of mental and physical problems later in life. Future studies on the impact of maternal parenting behavior on HCC should assess additional relevant factors for allostatic load, such as inflammatory processes in the course of child development. For instance, it should be investigated whether more inflammation in the mother is associated with more maternal role reversal and whether role/boundary confusion and/or inflammation or the interplay of both can explain the increased risk for mental and physical illness later in life. The findings of this study highlight the importance of early intervention programs that focus on the early interaction between mother and child. However, before too far-reaching conclusions are drawn on the implications of the observed findings for interventions, the results should be replicated in future studies with at risk families.

## Data Availability

The original contributions presented in the study are included in the article/Supplementary Material, further inquiries can be directed to the corresponding author/s.
